# MicroRNA Profile of Mouse Adipocyte-Derived Extracellular Vesicles

**DOI:** 10.3390/cells13151298

**Published:** 2024-08-01

**Authors:** Tamás Röszer

**Affiliations:** Department of Pediatrics, Faculty of Medicine, University of Debrecen, 4032 Debrecen, Hungary; roszer.tamas@med.unideb.hu

**Keywords:** obesity, fat, adipose tissue, endocrinology, metabolism

## Abstract

The post-transcriptional control of gene expression is a complex and evolving field in adipocyte biology, with the premise that the delivery of microRNA (miRNA) species to the obese adipose tissue may facilitate weight loss. Cells shed extracellular vesicles (EVs) that may deliver miRNAs as intercellular messengers. However, we know little about the miRNA profile of EVs secreted by adipocytes during postnatal development. Here, we defined the miRNA cargo of EVs secreted by mouse adipocytes in two distinct phases of development: on postnatal day 6, when adipocytes are lipolytic and thermogenic, and on postnatal day 56, when adipocytes have active lipogenesis. EVs were collected from cell culture supernatants, and their miRNA profile was defined by small RNA sequencing. The most abundant miRNA of mouse adipocyte-derived EVs was mmu-miR-148a-3p. Adipocyte EVs on postnatal day 6 were hallmarked with mmu-miR-98-5p, and some miRNAs were specific to this developmental stage, such as mmu-miR-466i-5p and 12 novel miRNAs. Adipocytes on postnatal day 56 secreted mmu-miR-365-3p, and 16 miRNAs were specific to this developmental stage. The miRNA cargo of adipocyte EVs targeted gene networks of cell proliferation, insulin signaling, interferon response, thermogenesis, and lipogenesis. We provided here a database of miRNAs secreted by developing mouse adipocytes, which may be a tool for further studies on the regulation of gene networks that control mouse adipocyte development.

## 1. Introduction

Excess development of adipose tissue leads to overweight and obesity, which eventually exacerbates chronic, incurable metabolic diseases and immune disorders, such as diabetes and cardiovascular, liver, and kidney diseases [1]. Today, there is a global escalation of obesity, which is mirrored by a rapid increase in the prevalence of obesity comorbidities [1]. The number and metabolic traits of adipocytes are key determinants of the adipose tissue volume and, eventually, obesity status [2]. An increase in preadipocyte proliferation leads to an increase in the adipocyte number, often termed as “adipocyte hyperplasia”—a permanent, irreversible expansion of fat depots. Increased lipogenesis leads to fat storage, decreases systemic energy expenditure, and leads to the expansion of the adipocyte volume, causing adipocyte hypertrophy. Adipocyte hypertrophy may be reverted by increased lipolysis and fatty acid oxidation, often associated with thermogenesis in the process of uncoupled mitochondrial respiration in adipocytes [3].

Nutritional signals, hormones, and adipokines are the key signals that control these key mechanisms and determine adipocyte proliferation and the balance between lipogenesis, lipolysis, and thermogenesis. More recently, several small non-coding RNA species, the so-called microRNA (miRNA) species, have been identified in obesity that may interfere with the post-transcriptional control of gene networks associated with preadipocyte proliferation and development [4,5], although the efficacy of the miRNA-controlled cellular functions needs better validation [6].

The post-transcriptional control of gene expression is a complex and evolving field in adipocyte biology, with the premise that the delivery of miRNA species to the obese adipose tissue may help weight loss [7,8]. Adipocytes shed extracellular vesicles [6,9], and extracellular vesicles carry miRNA species [6]. These miRNA species remain relatively stable in the bloodstream since the membrane layer of the extracellular vesicles shields the RNA cargo from extracellular RNases [10,11]. The miRNA species within the extracellular vesicles hence may function as intercellular messengers [12,13,14]. Moreover, various adipose-tissue-derived miRNAs may serve as biomarkers for obesity-associated disease risk assessment [15,16]. Some extracellular-vesicle-bound microRNAs that are circulating in the bloodstream may be specific hallmarks of various stages of obesity or may be indicators of adipose tissue inflammation [16], the degree of adipocyte thermogenesis [17], and adipose tissue fibrosis [18].

While obesity-associated miRNA species are in the spotlight of scientific interest today, we know little about the miRNA profile of adipocytes under physiological conditions, especially during adipocyte development. In mice, for instance, the subcutaneous fat depot begins to expand after birth, with a prevalence of thermogenic adipocytes during the suckling period [19,20]. After weaning, there is a gradual loss of lipolytic and thermogenic fat cells, and the fat depot is dominated by fat-accumulating adipocytes that have strong lipogenic metabolic activity [20,21,22]. These two developmental stages of the mouse adipose tissue are associated with two distinct functional states of adipocytes. In obesity management, it is a desirable aim to shift adipocytes from lipogenesis toward lipolysis and thermogenesis. Hence, comparing the miRNA profile of young and adult mouse adipocytes may identify mechanisms that allow the metabolic shift from fat storage to fat breakdown.

Physiological adipose tissue development requires a controlled sequence of proliferative, thermogenic, and lipogenic stages [22], and it is plausible that these developmental stages have their own specific miRNA expression patterns. However, we know little about the developmental-stage-specific miRNA profile of adipocytes. Here, we aimed to define the miRNA cargo of extracellular vesicles secreted by young and adult mouse adipocytes. Young mouse adipocytes are mostly thermogenic and lipolytic, while their adult counterparts have limited thermogenic competence and they accumulate fat [22]. Knowing the miRNA profiles associated with these two distinct functional stages of adipocytes may provide insight into the regulation of gene networks that control mouse adipocyte development.

## 2. Materials and Methods

### 2.1. Adipocyte Isolation and In Vitro Culture

We used C57/BL6J male mice at the age of postnatal day 6 (P6) and day 56 (P56), bred under specific-pathogen-free conditions. The inguinal adipose tissue depots were dissected under a sterile hood, and preadipocytes of the stromal vascular fraction (SVF) were isolated following the collagenase digestion of the adipose tissue extracellular matrix, as described [23,24,25]. To ensure the depletion of adipose tissue macrophages (ATMs) from the harvested preadipocytes, we used magnetic bead cell purification of the SVF with an antibody against the F4/80 antigen (Miltenyi Biotec, Bergisch Gladbach, Germany) [26]. Preadipocytes were maintained in high-glucose Dulbecco’s modified Eagle medium (DMEM) supplemented with 1% penicillin–streptomycin, 1% L-glutamine, and 20 μg/mL of insulin (all from Merck, Darmstadt, Germany), as described [19] (Supplemental Figure S1). To avoid contamination with bovine EVs, we supplemented the cell culture media with EV-depleted fetal calf serum (Gibco, Grand Island, NY, USA) throughout the study.

### 2.2. Isolation of Extracellular Vesicles from Cell Culture Media

We seeded the isolated preadipocytes into 50 mL culture flasks. Each flask was seeded with preadipocytes pooled from 6–8 mice at age P6 (depending on the litter size) and 3 mice at age P56, as shown in the summary scheme in Supplemental Figure S1. SVF cells were pooled from multiple animals to obtain sufficient SVF cells for adipocyte differentiation. Once the cells reached 70% confluence, adipocyte differentiation was induced by maintaining preadipocytes in 10 mL of high-glucose DMEM with 1% penicillin–streptomycin, 1% L-glutamine, 20 μg/mL of insulin, 50 mM 3-isobutyl-1-methylxanthine, 1 mM dexamethasone, and 1 mM rosiglitazone. This was followed by the purification of extracellular vesicles (EVs) from the cell culture media. Following 18 h of incubation, 10 mL of the cell culture media from each flask was collected, and EVs were precipitated with the EPStep exosome precipitation solution (Immunostep, Centro de Investigación del Cáncer, Campus Miguel de Unamuno, Salamanca, Spain) and concentrated by ultracentrifugation to obtain ~5 μL of wet pellets from each flask. EVs were analyzed with FACS using capture beads and labeling for CD63 (Immunostep) and were processed for flow cytometry. EV pellets of 3–3 flasks from P6 and P56 cultures were used to extract RNA (Supplemental Figure S1). The nucleic acid content of the EVs was labeled with Sytox Green (Thermo Fisher, Waltham, MA, USA). Endocytosis by means of pinocytosis was assessed by incubating adipocytes with FITC-conjugated dextran, followed by FACS analysis (BD LSR II) or fluorescence microscopy. Fractions of EV pellets and adipocytes were also fixed in paraformaldehyde/glutaraldehyde and processed for transmission electron microscopy (TEM) analysis, as described [24]. The morphology of EVs was analyzed with conventional TEM [27]. The EV diameter and area were measured with ImageJ 1.51 (NIH) with manual annotation, as described [28,29].

### 2.3. miRNA Detection

Small RNA species were isolated from the EV pellets using the Quick-DNA/RNA Miniprep kit for miRNA extraction (Zymo Research, Irvine, CA, USA), followed by quality control performed on an Agilent 2100 device to determine the RNA quantity and integrity and agarose gel electrophoresis to control sample purity. The RNA yield was 5.4–5.8 ng/μL, with integrity values of 2.7–2.9. Quality control, library preparation, and sequencing were performed by Novogene Co., Ltd. (Beijing, China). For library construction, 3′ and 5′ adaptors were ligated to the 3′ and 5′ ends of small RNAs, respectively. First-strand cDNA was synthesized after hybridization with a reverse transcription primer. A double-stranded cDNA library was generated through PCR enrichment. After purification and size selection, libraries with insertions between 18 and 40 bps were used for Illumina sequencing with SE50. The library was checked with Qubit and real-time PCR (using 18 cycles) for quantification and a bioanalyzer for size distribution detection (Supplemental Figure S2). Raw data in FASTQ format were first processed through custom Perl and Python scripts. In this step, clean reads were obtained by removing reads containing ploy-N, with 5′ adapter contaminants, without the 3′ adapter or the insert tag, or containing ploy A or T or G or C and low-quality reads from raw data. At the same time, Q_20_, Q_30_, and GC contents of the raw data were calculated. The small RNA tags were mapped to the reference sequence by Bowtie. In the next step, we used miRBase to identify precursor and mature sequences of the candidate miRNA species [30]. The characteristics of the hairpin structure of miRNA precursors were used to predict novel miRNAs. For novel miRNA prediction, we used miREvo [31] and mirdeep2 [32] by exploring the secondary structure, the Dicer cleavage site, and the minimum free energy of the small RNA tags.

miRNA expression levels were estimated by transcripts per million, as described [33]. Differential expression analysis of P6 and P56 samples was performed using the DESeq R package (1.8.3). The *p*-values were adjusted using the Benjamini and Hochberg method. The corrected *p*-value of 0.05 was set as the threshold for significantly differential expression [34].

Candidate miRNA targets were identified by using TargetScan screens. For the analysis, TargetScan (release 8.0) was used [30]. The predictions were ranked according to the predicted targeting efficacy using cumulative weighted context scores (CWCSs) of sites, with lower values indicating a higher probability of targeting through a specific miRNA. Gene ontology and pathway enrichment analyses of the identified transcripts were performed by Enrichr [35]. For the analysis, a CWCS cutoff was set at −0.2 to reduce the probability of false-positive connections and to keep a manageable number of genes to be analyzed further. We used Enrichr for gene ontology (GO) analysis, including the involvement in biological processes, as well as the molecular function and cellular location of gene products. The resulting bar graphs were sorted by the combined *p*-value ranking. Further, interactome mapping of miRNA target genes was carried out via the Search Tool for the Retrieval of Interacting Genes/Proteins (STRING) (v12.0) [36]. Several proteins in the center of the generated network were examined in more detail by using the included web interface with access to data that provided an overview of the proteins and their functions. In addition, type I, type II, and type III interferon (IFN)-regulated target genes were identified by using Interferome (v2.01) [37]. Venn diagrams showing the number of genes regulated by one or more IFN type were generated. Small RNA sequencing data are available in GEO, under accession numbers GSE191256, GSE193589, and GSE193590. Adipose tissue expression levels of specific genes were determined by secondary analysis of new-generation sequencing data [19,22], deposited in GEO, under accession numbers GSE185317 and GSE133500. In brief, the total tissue RNA was extracted by TRIzol reagent (Invitrogen, Carlsbad, CA, USA) according to the manufacturer’s instructions and was quantified using the NanoDrop™ 8000 Spectrophotometer (Thermo Fisher Scientific, Waltham, MA, USA), yielding 500–900 ng/μL of RNA from each sample. NGS analysis was performed by BGI Genomics (Beijing, China), as described, comparing samples from 3–3 mice at P6 and P56 [19,22]. Gene expression levels were compared with Student’s 2-tailed unpaired *t*-test.

## 3. Results

### 3.1. Morphology of Mouse Adipocyte Extracellular Vesicles

**Figure 1:**
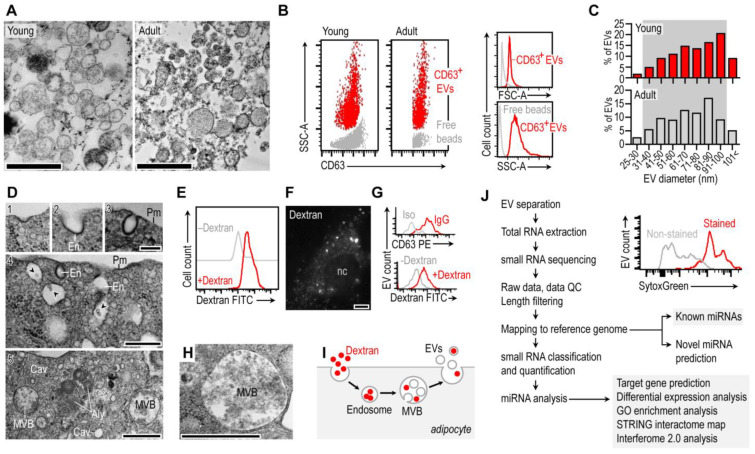
Mouse adipocytes shed EVs in the endosomal pathway. (**A**) TEM image of EVs released by young (postnatal day 6) and adult (postnatal day 56) mouse adipocytes in vitro. Scale: 1 μm. Representative images from three independent EV pellets. (**B**) FACS analysis of EVs secreted by young and adult mouse adipocytes; representative histograms from three assays. Free beads: remainder of capture beads used to enrich EVs. (**C**) Size distribution of adipocyte EVs using TEM images of three independent EV pellets. Gray background labels show the expected size range for EVs. (**D**) Transmission electron microscopy images of mouse adipocytes, cultured in vitro, showing clathrin-coated pits, endosome budding (1–4), and multivesicular bodies (MVBs) (5). Pm: plasma membrane; En: endosome; Aly: autolysosome; Cav: caveolae; arrowhead: extracellular vesicles (EVs) in MVBs. Scale: 1 μm. (**E**) The endosomal pathway of EV generation was tested by incubating adipocytes with FITC-conjugated dextran, a marker of fluid-phase endocytosis (pinocytosis). Dextran is taken up by endosomes and may later accumulate in MVBs or in lysosomes. FACS analysis of mouse adipocytes cultured without FITC-conjugated dextran (–Dextran) or with FITC-conjugated dextran (+Dextran). (**F**) Fluorescence microscopy showing endocytosed FITC–dextran within adipocytes. nc: nucleus; scale: 10 μm. (**G**) EVs secreted by the dextran-incubated adipocytes were collected and analyzed further with FACS. Dextran was present in the EVs, showing that the endosomal pathway contributes to EV generation. (**H**) TEM image of an MVB; scale: 1 μm. (**I**) Scheme of the endosomal pathway of EV generation. (**J**) Workflow of the analysis of the miRNA cargo of EVs. The inset shows FACS analysis of the nucleic acid content of EVs. We used SytoxGreen to stain nucleic acids in EVs. Histogram showing SytoxGreen fluorescence intensity of EVs.

First, we isolated primary mouse preadipocytes from the inguinal fat depot of young C57/BL6 mice, differentiated them into adipocytes, and maintained them in vitro. Extracellular vesicles (EVs) that were secreted by the adipocytes were collected from the cell culture media and analyzed with TEM (Figure 1A, Figure S3, and S4). The majority of the EVs had a clear, electrolucent content, although EVs with various membrane inclusions, microvesicles, and electrodense content were also detectable (Figure 1A). The cell membrane of the adipocyte EVs expressed the exosomal marker CD63 (Figure 1B). Adipocytes secreted EVs that had a diameter between 30 and 100 nm (Figure 1C), which is within the expected size range of exosomes. The adipocyte morphology was further analyzed with TEM using in vitro differentiated adipocytes from both young and adult mice. We found morphological signs of inverse budding of the plasma membrane in both young and adult adipocytes, which is a hallmark of endosome formation (Figure 1D). Coherently, adipocytes were rich in endosomes and contained numerous multivesicular bodies (MVBs) (Figure 1D). MVBs are generated by the inverse budding of endosomes or during autophagy of the cellular content [38] and release EVs by exocytosis, following fusion with the cell membrane [29].

We next asked whether EVs of adipocytes were generated by the endosome–MVB route. Since endosomes mediate the cellular uptake of dextran particles, we incubated the adipocyte culture with FITC-conjugated dextran particles. These particles were readily taken up by the adipocytes, shown by the presence of FITC^+^ intracellular granules in adipocytes incubated with FITC-conjugated dextran. Both fluorescence-assisted cell sorting (FACS) analysis (Figure 1E) and fluorescent microscopy (Figure 1F) confirmed the uptake of FITC–dextran by both young and adult adipocytes. The fluorescent signal of the dextran particles eventually appeared in the CD63^+^ EVs secreted by the adipocytes (Figure 1G).

Adipocyte MVBs were filled with EVs (Figure 1H), and the presence of a dextran cargo in the EVs suggested that adipocytes generate EVs in the endosome–MVB pathway by the inverse budding of endosomes and the eventual exocytotic release of EVs by the fusion of MVBs with the cell membrane (Figure 1I).

The adipocyte EVs were positively stained for nucleic acids with SytoxGreen, suggesting the presence of nucleic acids within their cargo (Figure 1J, inset). As a next step, we collected EVs from the cell culture supernatants and processed them for total RNA extraction and small RNA sequencing (Figure 1J, Supplemental Table S1).

### 3.2. The Most Abundant miRNA in Mouse Adipocyte EVs Was mmu-miR-148a-3p

**Figure 2:**
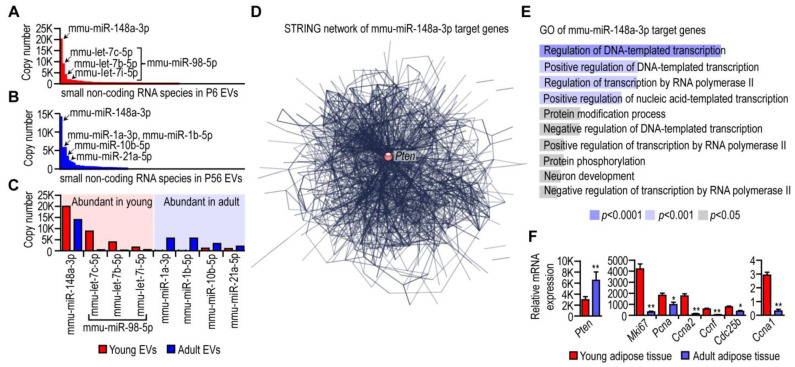
Abundantly expressed miRNA species in mouse adipocyte EVs. (**A**) Copy numbers of miRNA species in EVs secreted by young (postnatal day 6) mouse adipocytes. The highest-copy-number miRNA species are highlighted. (**B**) Copy numbers of miRNA species in EVs secreted by adult (postnatal day 56) mouse adipocytes, with highlighting of the most abundant miRNAs. (**C**) Comparison of average copy numbers of the most abundantly expressed miRNAs. Average copy numbers were determined by using small RNA sequencing data from 3–3 EV samples from young and adult mouse adipocyte cultures. (**D**) STRING network formed by target genes of mmu-miR-148a-3p. (**E**) Gene ontology (GO) terms of target genes of mmu-miR-148a-3p. (**F**) Transcript levels of *Pten* and cell-cycle-regulating gene products in young and adult mouse adipose tissue. Next-generation sequencing data. ** *p* < 0.01, * *p* < 0.05, Student’s 2-tailed unpaired *t*-test.

Following the small RNA sequencing of the RNA cargo of young and adult adipocyte EVs, we found that mmu-miR-148a-3p is the most abundant miRNA species in mouse adipocytes (Figure 2A,B). It was the most prevalent miRNA in both young and adult adipocyte EVs, albeit with a lower copy number in adult adipocyte EVs (Figure 2C). This miRNA had 577 predicted target genes (Supplemental Table S2), and these potential target genes formed a single, one-cluster gene network (Figure 2D). This gene network translated into a protein–protein interaction network with the tumor suppressor phosphatase and tensin homolog (PTEN) as a central hub (Figure 2D). Gene ontology analysis of the mmu-miR-148a-3p target genes indicated their role in gene transcription control, and many members of the target gene network were associated with cell cycle progression (Figure 2E). The level of mmu-miR-148a-3p was higher in young adipocyte EVs, and *Pten* mRNA was lower in young adipocytes than in their adult counterparts (Figure 2F).

Cell-cycle-associated target genes of PTEN included cyclin-dependent kinase 8 (*Cdk8*), cyclin-dependent kinase 5 regulatory subunit 1 (p35, *Cdk5r1*), cyclin-dependent kinase 19 (*Cdk19*), cyclin F (*Ccnf*), cyclin-dependent kinase 13 (*Cdk13*), cyclin A2 (*Ccna2*), cell division cycle 14A (*Cdc14a*), and cell division cycle 25B (*Cdc25b*). Adipocyte progenitors in mouse inguinal fat have the potential to enter the cell cycle and eventually lead to adipose tissue hyperplasia [39]. Knockdown of PTEN enhances the cell proliferation and differentiation of adipocyte progenitors in mouse [40], and an increase in the mmu-miR-148a-3p level is associated with increased bone marrow adiposity in humans [41]. Accordingly, we found a prevalent expression of cell-proliferation-associated genes in young adipose tissue, such as *Mki67*, *Pcna*, *Ccna1*, *Ccna2*, *Cdc25b*, and *Ccnf* (Figure 2F). Altogether, mmu-miR-148a-3p was an abundantly expressed miRNA species in mouse adipocyte EVs, and its higher copy number was concomitant with the hallmarks of cell proliferation in young adipose tissue. The miRNA composition of both young and adult adipocyte-derived EVs was distinct from the miRNA profile of mesenchymal-stem-cell-derived EVs [42,43].

### 3.3. Developmental-Stage-Specific miRNA Cargo of Mouse EVs

**Figure 3:**
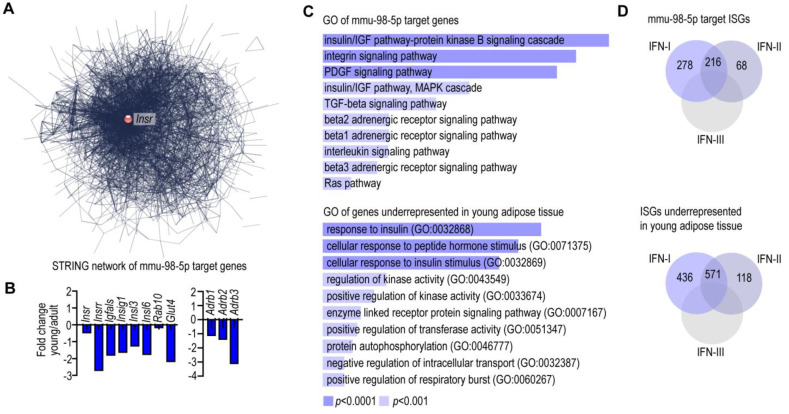
EVs of young mouse adipocytes secrete mmu-miR-98-5p. (**A**) STRING network showing an interactome map of mmu-miR-98-5p target genes. (**B**) Comparison of the transcript levels of insulin receptor (*Insr*), insulin signal pathway gene products, and beta-adrenergic receptors in young and adult mouse adipocytes using NGS data. (**C**) GO analysis of mmu-miR-98-5p target genes. (**D**) Venn diagrams summarizing the numbers of interferon-stimulated genes (ISGs) that are targets of mmu-miR-98-5p (Top). Numbers of ISGs underrepresented in young mouse adipocytes using NGS data [22] (Bottom). IFN-I: type I IFN response; IFN-II: type II IFNs; IFN-III: type III IFNs.

As a next step, we compared the miRNA expression pattern of EVs secreted by young and adult mouse adipocytes. We found that mmu-miR-98-5p is the second-most abundant miRNA species—following mmu-miR-148a-3p—in EVs secreted by young adipocytes (Figure 2A,C). This miRNA species involves three sequences that appeared separately in our sequence analysis: mmu-let-7c-5p, mmu-let-7b-5p, and mmu-let-7i-5p (Figure 2A). Their expression levels were significantly higher in young adipocyte EVs than in their adult counterparts (*p* < 0.0001). This miRNA species had 1046 potential target genes, which formed a uniform gene network, with insulin receptor (*Insr*) being the central node (Figure 3A). Accordingly, the expression level of *Insr* was lower in young adipocytes than in their adult counterparts (Figure 3B). Moreover, components of the insulin signaling pathway were also less expressed in young adipocytes than in adult adipocytes: *Insrr*, encoding insulin-receptor-related receptor; *Igfals*, encoding insulin-like growth-factor-binding protein, acid-labile subunit; *Insig1*, encoding insulin-induced gene 1; *Insl3*, encoding insulin-induced gene 3; *Insl6*, encoding insulin-induced gene 6; Rab10, encoding Ras-related protein 10; and *Glut4*, encoding glucose transporter 4 (Figure 3B).

Gene ontology analysis of mmu-miR-98-5p target genes also indicated their association with insulin signaling and insulin response (Figure 3C) and, moreover, with beta adrenergic receptor signaling (Figure 3C). Coherently, the transcript levels of genes encoding beta adrenergic receptors (*Adrb1*, *Adrb2*, *Adrb3*) were lower in young adipocytes than in their adult counterparts (Figure 3B). Moreover, mmu-miR-98-5p had 560 interferon-stimulated genes (ISGs) as putative targets (Figure 3D). Young adipocytes had a general suppression of IGSs (Figure 3D), as shown previously [21].

**Figure 4:**
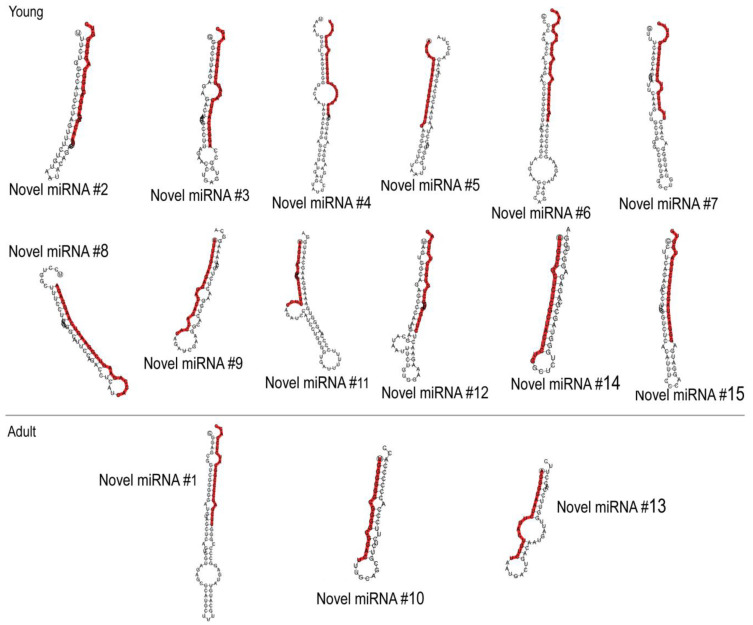
Sequences and predicted structures of novel miRNAs of mouse adipocyte EVs. Novel miRNA species have been named according to their order of identification during sequencing. (**Top**): Novel miRNAs in EVs secreted by young mouse adipocytes. (**Bottom**): Novel miRNAs secreted in EVs by adult mouse adipocytes.

**Figure 5:**
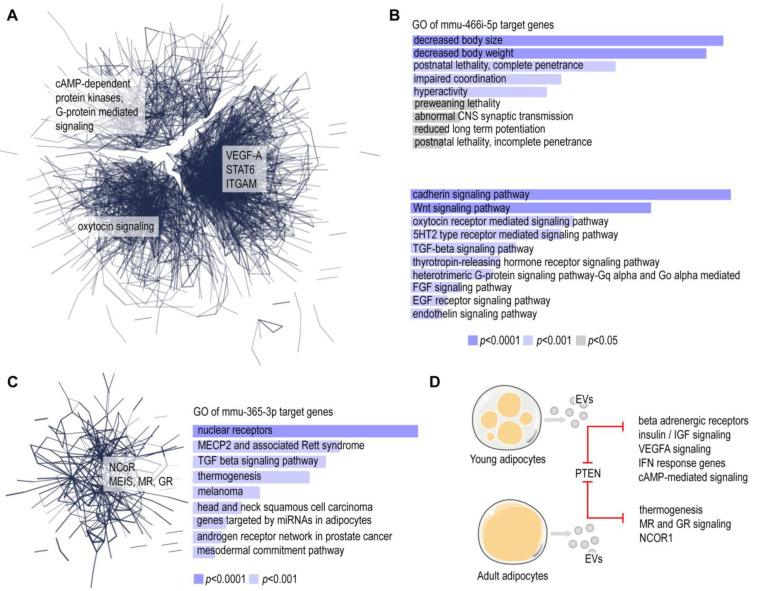
Possible targets of miRNAs specific to young and adult adipocyte EVs. (**A**) STRING network of target genes of mmu-miR-466i-5p. This miRNA was present only in young adipocyte EVs—lacking in adult adipocyte EVs—and had the highest copy number among miRNAs that were specific to young adipocyte EVs. (**B**) GO enrichment analysis of mmu-miR-466i-5p. (**C**) STRING network and GO enrichment analysis of target genes of mmu-miR-365-3p. (**D**) Proposed molecular targets of miRNAs secreted in adipocyte EVs.

We identified 15 novel miRNAs (Figure 4). There were 12 novel miRNA species that were specific to EVs of young adipocytes and only 3 that were specific to adult adipocytes (Figure 4, Supplemental Table S1). As a next step, we identified 212 miRNA species that were specific to EVs of young adipocytes and were lacking in EVs of adult adipocytes (Supplemental Table S3). Among these miRNAs, mmu-miR-466i-5p had the highest copy number. This miRNA had 3789 potential target genes, containing a total of 7307 complementary sites (Supplementary Table S4). This was followed by mmu-miR-5126, with 253 potential target genes; mmu-miR-3473b, with 3857 target genes; and mmu-miR-483-5p, with 672 target genes (Figure 5A). Target pathways included STAT6, VEGF-A, ITGAM, oxytocin signaling, cAMP-dependent protein kinases, and G-protein-mediated signaling. Gene ontology analysis indicated a general association of the putative target genes with body growth, nervous system development, and cadherin and Wnt signaling (Figure 5B).

Lastly, we identified only 16 miRNA species that were specific to the EVs of adult adipocytes, i.e., they were lacking in the EVs of young adipocytes, while being present in the EVs of adult adipocytes (Supplemental Table S1). These miRNAs were mmu-let-7b-3p, mmu-miR-187-3p, mmu-miR-190b-5p, mmu-miR-1969, mmu-miR-365-3p, mmu-miR-3960, mmu-miR-450b-5p, mmu-miR-467a-3p, mmu-miR-467d-3p, mmu-miR-493-5p, mmu-miR-494-3p, mmu-miR-5108, mmu-miR-677-5p, mmu-miR-7084-5p, mmu-miR-7a-1-3p, and mmu-miR-802-5p. Among these, the most abundant miRNA species was mmu-miR-365-3p. This miRNA had a small target gene network formed by a cluster of genes associated with nuclear receptor corepressor (NCoR), homeobox protein Meis1 (MEIS), mineralocorticoid receptor (MR), and glucocorticoid receptor (GR) signaling (Figure 5C). Coherently, gene ontology analysis indicated the association of this gene network with nuclear receptor signaling, TGFβ signaling, and thermogenesis (Figure 5C).

## 4. Discussion

Here, we provided a database of miRNA species that are secreted by mouse adipocytes in EVs. Our aim was to describe the miRNA secretome during mouse adipocyte development, especially to prove the functions of the miRNA cargo of adipocyte EVs. Indeed, the versatility of the interactions between miRNAs and their mRNA targets leads to possible interference with the transcription of hundreds or even thousands of genes, making them regulators of gene networks rather than individual genes. Here, we defined those gene networks that are potential targets of the identified miRNAs in mice.

Irrespective of their developmental stage, mouse adipocyte EVs contain mmu-miR-148a-3p abundantly. This miRNA is a predicted tumor suppressor in various cell types [44,45]. Its association with adipocyte-derived stem cells has also been shown before, and its level increases during adipocyte differentiation [46] and is associated with adiposity [41,46]. Its abundance in adipocyte EVs suggests that it may help to improve the identification of adipocyte-derived EVs in mouse plasma, and changes in its plasma level might reflect the adipose tissue volume. We found that a *Pten*-centered gene network is the most probable target of mmu-miR-148a-3p. Young adipocyte EVs had a higher mmu-miR-148a-3p copy number than their adult counterparts. Coherently, we found suppressed *Pten* mRNA expression in young adipocytes. While we did not present here experimental evidence on the function of mmu-miR-148a-3p in adipocytes, previously shown targets of mmu-miR-148a-3p include Krüppel-like factor 4 (KLF4) and the mesenchymal–epithelial transition factor c-MET. Both are regulated by PTEN. KLF4 is physically associated with PTEN, and their dissociation in response to TGFβ stimulation leads to the transcriptional activation of KLF4 [47]. Moreover, PTEN upregulates KLF4 expression [48]. PTEN also mitigates c-MET signaling [49].

In adipocytes, KLF4 activates adipogenesis [50]. However, the level of KLF4 mRNA and protein is lower in the omental adipose tissue of obese individuals than in lean individuals [51]. Similarly, high-fat-diet feeding reduces the adipose tissue KLF4 mRNA and protein level in mice [51]. Similar to KLF4, c-MET appears to promote adipocyte differentiation [52]. PTEN mutations are associated with obesity and with a paradoxically enhanced insulin sensitivity [53]. It is plausible, although still to be proven, that the suppression of *Pten* expression by mmu-miR-148a-3p may function as a counter-regulatory mechanism of adipogenesis and protects adipocytes from lipid overload. Since PTEN inhibits cell cycle progression, a reduced *Pten* level in young adipocytes may be indicative of ongoing cell proliferation. This possibility is supported by the higher transcript levels of key cell cycle genes in young adipocytes. Altogether, it is plausible that mmu-miR-148a-3p may be associated with cell cycle control and lipogenesis in mouse adipocytes. As adipocyte EVs may be spread by the bloodstream, they may also target distant cells, such as hepatocytes and skeletal muscle cells. In the liver, PTEN ablation causes fatty acid synthesis and fatty liver [54]. In the skeletal muscle, inhibition or a lack of PTEN mitigates muscle dystrophy and increases muscle mass [55,56].

In the EVs of young adipocytes, mmu-miR-98-5p was the most abundant miRNA species, following mmu-miR-148a-3p. The function of miR-98-5p has been explored in in vitro assays, showing that the overexpression of miR-98-5p decreases the transcript levels of both gluconeogenic and lipogenic genes in HepG2 cells, which may reduce lipid accumulation in hepatocytes [57]. Moreover, miR-98-5p has a pro-apoptotic effect and may inhibit osteogenic differentiation and mouse osteoblast growth in vitro [58]. We found that the target gene network of mmu-miR-98-5p is associated with insulin signaling, which was mirrored by reduced transcription of insulin-responsiveness genes in young adipocytes. Insulin receptor expression increases during adipocyte maturation [59,60], and insulin signaling is necessary for the expression of adipogenic transcription factors and, eventually, for the buildup of fat stores [61]. Altogether, the abundance of this miRNA in young adipocyte EVs suggests that it might be involved in the maintenance of a preadipocyte-like expression level of insulin receptors, albeit this possibility needs verification by testing the effect of this miRNA on mouse adipocytes. Moreover, mmu-98-5p targeted IFN response genes (ISGs), and coherently, the expression of ISGs was repressed in young adipocytes [21]. This, and earlier findings, suggests that mmu-miR-98-5p may impede pro-inflammatory signaling [62]. Adipocyte maturation and adipocyte hypertrophy in obesity are associated with an increase in pro-inflammatory gene expression, such as the increased expression of the target genes of nuclear factor kappa B [63] and IFN regulatory factors [64]. Hence, it is plausible that the miRNA secretome of young adipocytes may contribute to the suppression of pro-inflammatory genes, and this effect might appear within the adipose tissue or may be apparent in distant tissues exposed to adipocyte-derived EVs.

Functions of the miRNAs that are specific to young adipocytes, such as mmu-miR-466i-5p and mmu-miR-5126, are still to be defined. Limited evidence suggests that they are involved in inflammation control and the response to parasite infections [65,66,67]. Moreover, our STRING analysis showed that key genes that control adipose tissue development are potential targets of both miRNAs. These target genes are *Vegfa*, encoding VEGF-A; *Itgam*, encoding integrin alpha M; *Stat6*, encoding signal transducer and activator of transcription 6; *Oxtr*, encoding oxytocin receptor; and genes associated with protein kinase A signaling. The reduced expression of some of these genes, such as of *Stat6* and *Itgam*, protects from adipocyte hypertrophy. The lack of STAT6 makes mice resistant to diet-induced obesity, and their adipocytes do not develop hypertrophy under high-fat-diet feeding [68]. *Itgam*, along with other integrin family members and extracellular components, has increased transcription in obese adipose tissue [69]. Adipogenesis and thermogenesis are under the control of cAMP-dependent protein kinases [70].

The thermogenic potential of mouse adipocytes emerges on postnatal days 6 and 7 and gradually disappears at weaning age [19,20]. Adipocyte thermogenesis supports the maintenance of the core body temperature in newborn mammals and increases energy expenditure and eventually reduces lipid storage [71]. Some of the target genes of mmu-miR-466i-5p are involved in the control of adipocyte thermogenesis, such as *Oxtr* and *Vegfa*. Oxytocin reduces body adiposity and also prevents the generation of thermogenic fat cells in the process of the so-called adipocyte browning [72,73]. The overexpression of VEGF-A in mouse adipocytes triggers angiogenesis in the adipose tissue, along with an increase in thermogenic—beige—adipocyte numbers [74,75].

Adipocytes after birth actively build up lipid stores with simultaneously active lipolysis and thermogenesis [76]. Any imbalance between lipogenesis, lipolysis, and thermogenesis leads to metabolic disease. Excessive lipogenesis leads to obesity, while uncontrolled lipolysis and thermogenesis consume the energy reserves of the body, leading to wasting or cachexia [77]. It is plausible that young adipocytes secrete miRNAs that impede the excessive activation of thermogenesis, hence protecting the energy reserves of the adipose tissue. However, the premature development of the storage fat depot is also potentially inhibited by miRNAs secreted by young adipocytes. The latter mechanism involves the proliferation control of preadipocytes and the maintenance of preadipocyte-like metabolic traits (Figure 5D).

Adult adipocytes secrete miRNAs that target nuclear receptor signaling, including MR and GR signaling, as well as methyl-CpG-binding protein 2 (MECP2) signaling. The lack of MR signaling increases the transcription of genes associated with adipogenesis in mice [78]. Glucocorticoids, the endogenous ligands for GR, reduce thermogenic activity in the adipose tissue of mice [79]. However, the lack of GR increases insulin sensitivity and leads to adipose tissue expansion in mice [80]. Adipocyte MECP2 appears to increase the thermogenic potential and burning off of stored fat as heat [81]. Moreover, MECP2 protects against fatty liver in mice [82] and is necessary for muscle fiber growth and motor skills [83]. It is plausible that adult adipocyte EVs contain miRNAs that mitigate the thermogenic potential, favor lipid storage, and induce the deterioration of both liver muscle and skeletal muscle functioning.

## 5. Conclusions

Adipocyte differentiation is associated with profound changes in the expression patterns of gene networks, and adipocytes at various stages of postnatal development have distinct transcriptional landscapes. These global transcriptional differences reflect the functional state of the adipocytes. For instance, during obesity development, preadipocytes undergo a metabolic shift toward lipogenesis, which is often associated with cell proliferation and blunted thermogenic capacity of adipocytes. Here, we identified miRNAs that hallmark EVs derived from proliferating, lipolytic young adipocytes and lipid-storing adult adipocytes. These data highlight candidate miRNA species that may be used in future studies to alter the expression of gene networks rather than individual genes in adipocytes or in other metabolically relevant cells. This would allow changing the adipocyte developmental program and to better understand the post-transcriptional control of gene networks involved in obesity development.

## Data Availability

Raw sequencing data are available in the NIH GEO repository.

## Supplementary Materials

The following supporting information can be downloaded at https://www.mdpi.com/article/10.3390/cells13151298/s1: Figure S1: Workflow of cell culture and EV analysis; Figure S2: Quality control of libraries used for sequencing; Figure S3: Extracellular vesicles of young adipocytes; Figure S4: Extracellular vesicles of adult adipocytes; Table S1: Read counts of small RNAs in EVs; Tables S2–S4: miRNAs specific to young and adult adipocyte EVs, predicted target genes of miRNAs.
