# Supplementary material for: MicroRNA Profile of Mouse Adipocyte-Derived Extracellular Vesicles
Source: Cells. 2024 Aug 1;13(15):1298. doi: 10.3390/cells13151298 (PMC11311276; doi:10.3390/cells13151298)
Supplement: Supplementary file 1 [file cells-13-01298-s001.zip › R1 Supplement/Supplemental Figures 1-4.pdf]

Supplemental Figure 1. Workflow of cell culture and EV analysis

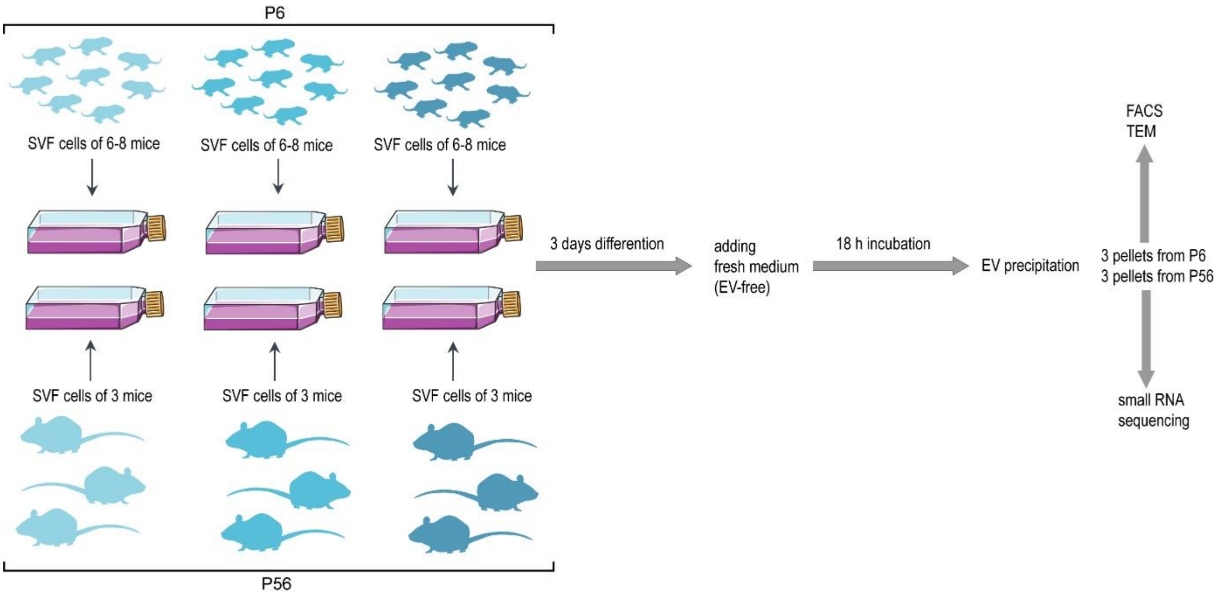

SVF: stromal vascular fraction
FACS: fluorescence activated cell sorting
TEM: transmission electron microscopy

Supplemental Figure 2. Quality control requirements for libraries used for sequencing

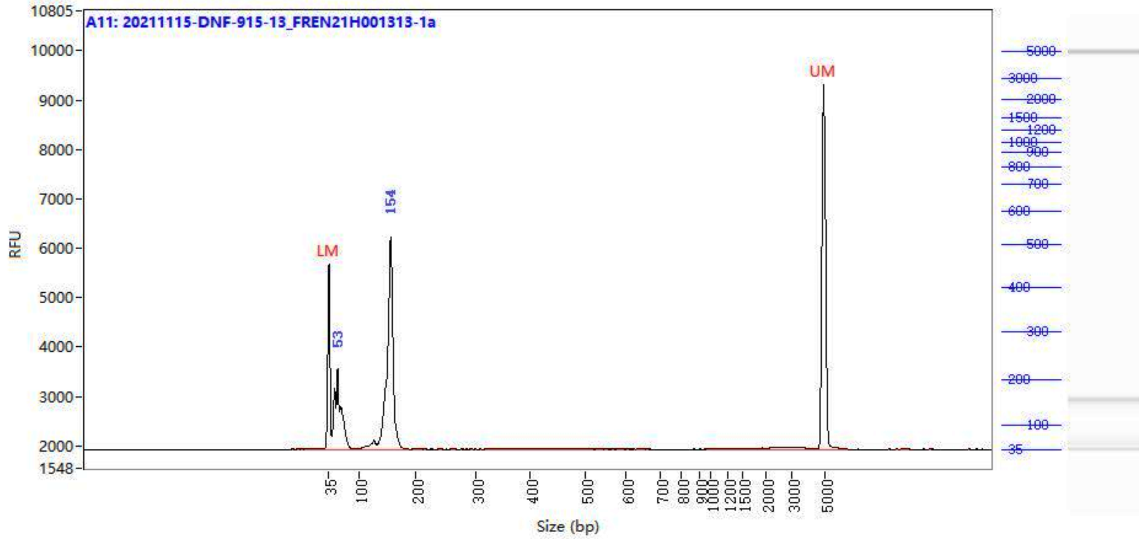

X-axis: Size (bp) reflects the size distribution of the sample in the capillary
Y-axis: RFU reflects the real-time fluorescence signal intensity of the sample during the capillary separation process, the higher the RFU value, the higher the sample concentration is
LM: low marker
The right side of the figure shows electrophoresis result with the corresponding size ranges.

Supplemental Figure 3. Extracellular vesicles of young adipocytes

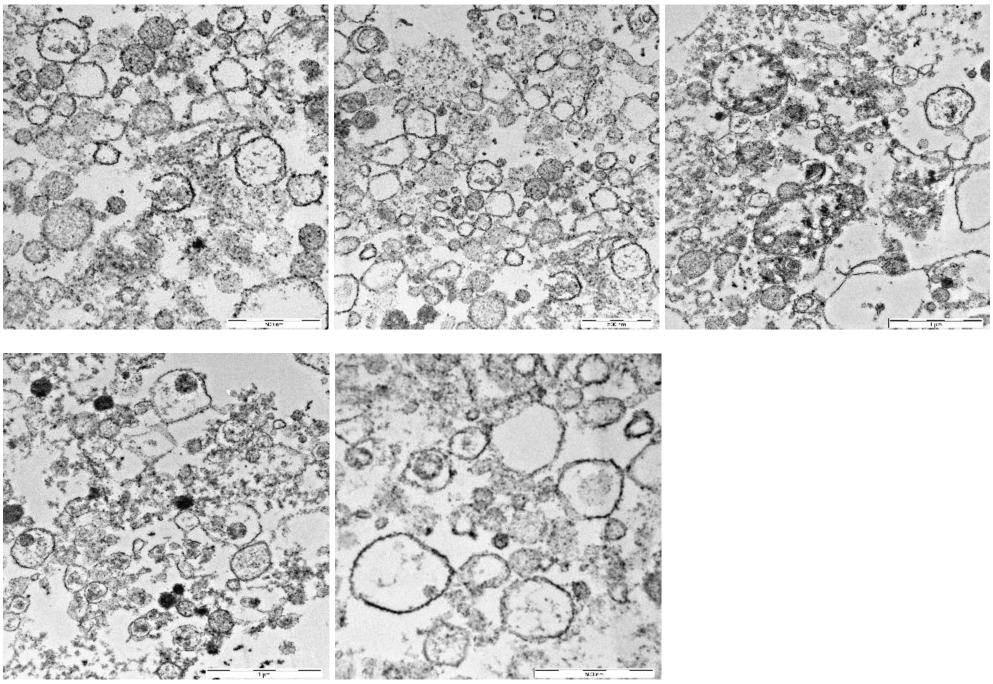

Supplemental Figure 4. Extracellular vesicles of adult adipocytes

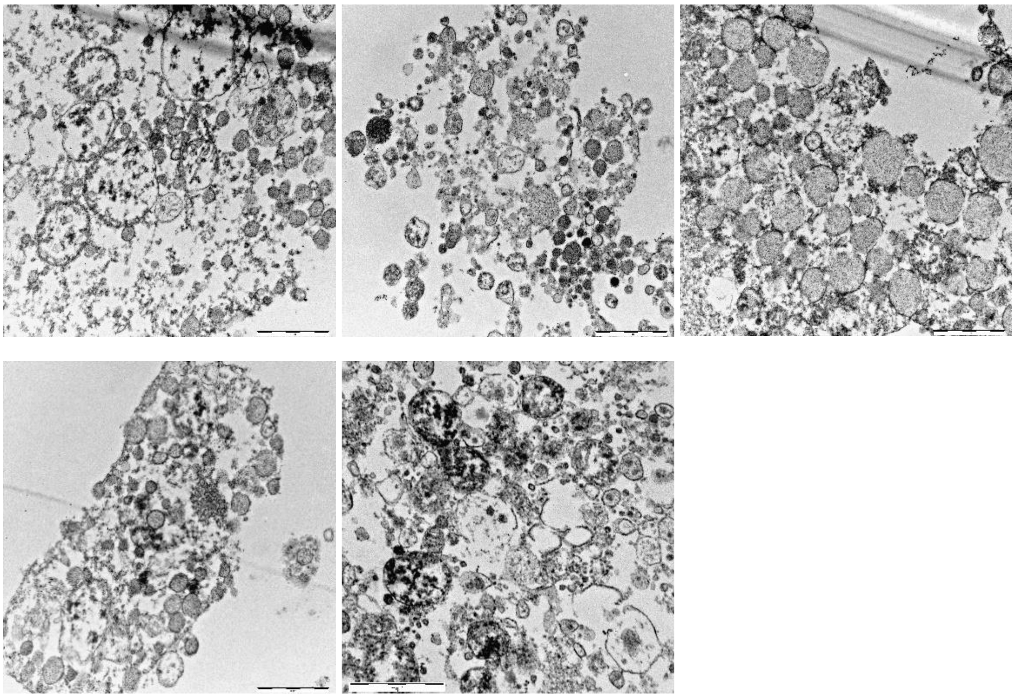

Figshare link to further images: doi 10.6084/m9.figshare.26363857
